# All for one: changes in mitochondrial morphology and activity during syncytial oogenesis^†^

**DOI:** 10.1093/biolre/ioac035

**Published:** 2022-02-14

**Authors:** Anna Z Urbisz, Łukasz Chajec, Karol Małota, Sebastian Student, Marta K Sawadro, Małgorzata A Śliwińska, Piotr Świątek

**Affiliations:** Faculty of Natural Sciences, Institute of Biology, Biotechnology and Environmental Protection, University of Silesia in Katowice, Katowice, Poland; Faculty of Natural Sciences, Institute of Biology, Biotechnology and Environmental Protection, University of Silesia in Katowice, Katowice, Poland; Faculty of Natural Sciences, Institute of Biology, Biotechnology and Environmental Protection, University of Silesia in Katowice, Katowice, Poland; Institute of Automatic Control, Silesian University of Technology, Gliwice, Poland; Faculty of Natural Sciences, Institute of Biology, Biotechnology and Environmental Protection, University of Silesia in Katowice, Katowice, Poland; Laboratory of Imaging Tissue Structure and Function, Nencki Institute of Experimental Biology of Polish Academy of Sciences, Warsaw, Poland; Faculty of Natural Sciences, Institute of Biology, Biotechnology and Environmental Protection, University of Silesia in Katowice, Katowice, Poland

**Keywords:** fusion, fission, nurse cell, oocyte, germ-line cyst

## Abstract

The syncytial groups of germ cells (germ-line cysts) forming in ovaries of clitellate annelids are an attractive model to study mitochondrial stage-specific changes. Using transmission electron microscopy, serial block-face scanning electron microscopy, and fluorescent microscopy, we analyzed the mitochondria distribution and morphology and the state of membrane potential in female cysts in *Enchytraeus albidus*. We visualized in 3D at the ultrastructural level mitochondria in cysts at successive stages: 2-celled, 4-celled, 16-celled cysts, and cyst in advanced oogenesis. We found that mitochondria form extensive aggregates—they are fused and connected into large and branched mitochondrial networks. The most extensive networks are formed with up to 10 000 fused mitochondria, whereas individual organelles represent up to 2% of the total mitochondrial volume. We classify such a morphology of mitochondria as a dynamic hyperfusion state and suggest that this can maintain their high activity and intensify the process of cellular respiration within the syncytial cysts. We found some individual mitochondria undergoing degradation, which implies that damaged mitochondria are removed from networks for their final elimination. As growing oocytes were shown to possess less active mitochondria than the nurse cells, the high activity of mitochondria in the nurse cells and their dynamic hyperfusion state are attributed to serve the needs of the growing oocyte. In addition, we measured by calorimetry the total antioxidant capacity of germ-line cysts in comparison with somatic tissue, and it suggests that antioxidative defense systems, together with mitochondrial networks, can effectively protect germ-line mitochondria from damage.

## Teaser

In the syncytial germ-line cysts of annelid *Enchytraeus albidus*, functioning during oogenesis, mitochondria form extensive networks that serve the needs of the growing oocyte.

## Graphical Abstract

**Graphical Abstract:**
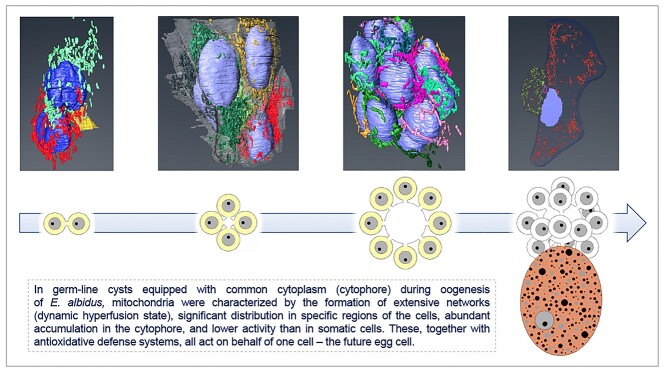

## Introduction

Oogenesis, a unique process leading to the formation of egg cells, requires many events that provide future eggs with all the necessary components that are subsequently used in embryo development. Oocytes, cells that start meiosis, undergo an intensive growth phase before they are ready for fertilization. Oocyte growth is usually divided into two phases: previtellogenesis when oocytes gather cell organelles and macromolecules (e.g., different classes of ribonucleoproteins (RNPs)) and vitellogenesis when oocytes become giant cells due to the accumulation of nutritive material [1–4]. This development of oocytes is complex and intensive, and it is believed that it requires a lot of energy [5, 6].

**Figure 1:**
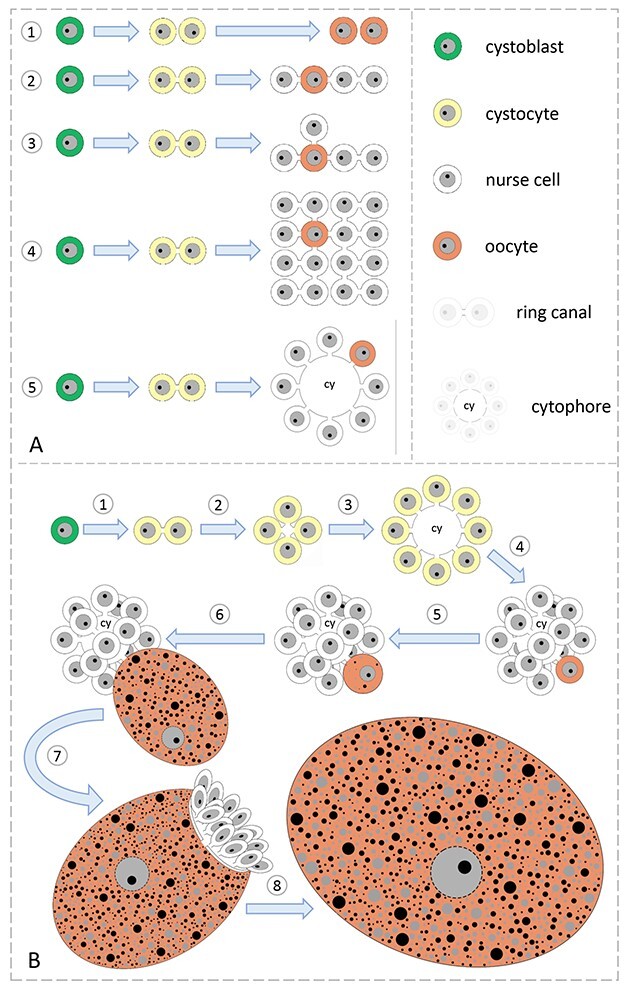
(A) Schematic diagram of two modes of oogonial divisions—complete mitoses and individual cells formation (1), and incomplete mitoses causing syncytial cyst formation of different architectures: (2) a linear cyst, in which all of the cells, except for the terminal ones, have two ring canals; (3) a linear cyst with branching sites, in which most of the cells form chains; occasionally some cells have three or more bridges; (4) branched cysts; the cystocytes have as many ring canals as the number of cell division rounds that have occurred; (5) cysts with a central and common cytoplasmic mass, in which each cell has one ring canal. (B) Formation and function of the cyst with common cytoplasm in *E. albidus* oogenesis. The germ cells undergo four rounds of mitosis, forming as follows: 2-celled cyst (1), 4-celled cyst (2), 8-celled cyst (3), and 16-celled cyst in which one cell differentiates into the oocyte, the other 15 into nurse cells (4). As oogenesis progresses, the oocyte enlarges significantly and gathers cytoplasm with cell organelles, macromolecules, and nutritive material; the growing oocyte is supplied by the nurse cells (5, 6). In late oogenesis, nurse cells and cytophore remnants are surrounded by the large vitellogenic oocyte and probably internalize into the oocyte cytoplasm (7). Finally, the oocyte becomes the individual cell—the egg (8).

In metazoans, oogenesis is associated with ongoing divisions of gonial cells (oogonia) occurring before meiosis. Two modes of such oogonial divisions are known: mitoses are complete, daughter oogonia are individual cells; or the mitotic divisions are not completed (the cytokineses are blocked), daughter cells are not fully separated and, as a consequence, form syncytial groups of cells—germ-line cysts (Figure 1A) [2, 7–11]. In cysts, all cells stay connected by cytoplasmic channels, termed intercellular bridges, cytoplasmic bridges, or ring canals, which are modified contractile rings [12–14]. Ring canals can reach 15 μm or more in diameter, allowing the transfer of cell components between interconnected cells [12, 13, 15, 16]. It should be mentioned here that the occurrence of germ-line cysts is not ubiquitous in oogenesis and, in some groups of animals, cysts have never been observed (e.g., in some insects, arachnids and annelids) [2, 17–19]. In contrast, in most insects, some nematodes, certain annelids, tardigrades, bryozoans, and numerous vertebrates, female germ-line cysts are formed [2, 11, 20–27]. The functioning of the female germ-line cysts varies among taxa. In some groups (e.g., *Xenopus*), cysts are transient, interconnected cells having the same morphology and fate—after cysts breakdown, all cells become oocytes [25]. In contrast, in numerous taxa (e.g., fruit fly, mice, *Caenorhabditis elegans*), the fates of clustered cells are different—some of them (sometimes only one) continue meiosis, gather nutrients, and become oocytes. The rest do not gather nutrients. Instead, they are very active in producing nutrients and transfer their cytoplasm toward oocyte(s). The latter cells are termed nurse cells and after serving their purpose die at the end of oogenesis [2, 24, 26, 28, 29].

The female germ-line cysts show high variability in the details of their organization (architecture). Several different systems have been identified (Figure 1A), but only a few of them are well described. One of the best-known examples is cysts functioning in *Drosophila melanogaster* ovaries (OV). Here, the cysts are formed by four rounds of synchronous and incomplete divisions and contain 16 cells, 15 nurse cells, and 1 oocyte. Due to the specific orientation of mitotic spindles, each interconnected cell possesses as many ring canals as the number of times it divides; this cyst architecture is termed maximally branched [8, 9, 11, 14]. In some arthropods, annelids, and vertebrates, sister cells are interconnected in a chain-like structure where, except for terminal ones, each cell has two ring canals or can form linear cysts with some branchings [2, 26, 30]. To summarize, the systems of cyst architecture described above, regardless of the number of cells and the number of ring canals, are based on the direct contact of a given cell with neighbors via one or more ring canals. A different system was found in clitellate annelids, echiurans, nematodes (with the best-known model being that of *C. elegans*), and mites. In these animals, the center of the cysts is occupied by a common and anuclear mass of cytoplasm of different shapes and dimensions. This cytoplasmic core is termed the cytophore (in annelids—clitellates and echiurans), rachis (in nematodes), or medulla (in mites) [21, 24, 28, 31–34]. As a rule, in germ cysts with a cytophore, each cell has one ring canal that connects it to the common cytoplasm (Figure 1A). This common cytoplasm is involved in the transfer of cell organelles between interconnected cells [24].

Although the organization of cysts and many aspects of their formation and functioning are becoming better understood and described (mainly in model organisms such as the fruit fly), an exciting and still unknown issue is the organization and functioning of mitochondria in such syncytial cell groups. It is generally accepted that mitochondria play a significant role in oogenesis by delivering the energy (by generating most of the adenosine triphosphate—ATP), which is indispensable for egg development, and by their inheritance for the future embryo [35–37]. Mitochondrial function during reproduction is correlated with sperm and oocyte growth and embryo development. Such aspects as mitochondrial morphology and distribution affect oocyte quality and successful embryo development [37]. According to the literature, mitochondria change their morphology throughout reproduction, including oogenesis [37].

The dynamism of mitochondria is related to two opposing processes—fusion and fission—that merge individual organelles into more extensive mitochondrial networks or split the networks into the individual mitochondria. The balance between these processes has implications for mitochondrial morphology, and their distribution throughout the cell, which in turn is directly linked to mitochondrial function [38–42]. Mitochondrial networks have been found in different cell types, and the molecular mechanisms of fusion/fission events are well known from model systems such as yeast and mammalian tissue cell cultures [43–48]. However, data devoted to the changes in mitochondrial morphology during oogenesis are very limited. One such report focused on mitochondrial networks during early oogenesis in the insect *Thermobia domestica*, where a single mitochondrial network was detected as a part of the characteristic organelle complex, called the Balbiani body [49]. Another example is our previous analysis of the mitochondrial morphology in syncytial cysts in OV of the white worm *Enchytraeus albidus* (representative of clitellate annelids) [50].

In *E. albidus* female cysts, germ cells differentiate into 1 oocyte and 15 nurse cells (Figure 1B and [23, 50, 51]). As a rule, each cell is connected via a single ring canal to the centrally located cytophore (Figure 1B and [23, 50]). A previous study by Urbisz and coauthors [50] revealed extensive mitochondrial networks (in the so-called dynamic hyperfusion state) in the analyzed nurse cells and the cytophore and within the cytoplasm filling ring canals. Thus, germ-line cysts formed in *E. albidus* oogenesis are a valuable model for studying stage-specific changes in mitochondrial morphology and distribution in germ-line syncytia.

This work presents the comprehensive results of analyses devoted to mitochondria, during cyst formation and functioning, using a wide set of methods, including light microscopy (LM), transmission electron microscopy (TEM), serial block-face scanning electron microscopy (SBEM), fluorescence, flow cytometry, and calorimetry. Our first goal was to analyze the mitochondrial morphology, which is a manifestation of their dynamism, and the distribution of mitochondria throughout the cyst, during the process of cyst formation. These analyses showed extensive networks of connected mitochondria in *E. albidus* germ-line cysts.

Changes in mitochondrial morphology and their connection into more prominent organelles (networks) is associated by some authors with more efficient ATP production, as was demonstrated in skeletal muscle, heart muscle, neurons, and astroglial cells [39, 41, 52–55]. The same is suggested for reproductive cells, in which it is believed that mitochondrial networks may produce more ATP than fragmented ones [37]. Therefore, the second goal was to investigate the level of mitochondrial activity in *E. albidus* germ-line cysts. We checked whether the level of mitochondrial connection is reflected in their activity, that is, whether a more extensive network means more active mitochondria.

Experimental studies on yeast or cultured cells show the relationship between mitochondrial fusion and fission and cellular stress [38, 39, 56]. Prolonged stress may lead to the accumulation of mitochondrial damage and depolarization, resulting in mitochondrial fragmentation. Dysfunctional mitochondria containing the mutant genomes may lose potential and become degraded by mitophagy. However, upon cellular stress or starvation, the mitochondria undergo hyperfusion, joining into extensive mitochondrial networks. Researchers suggest that hyperfusion may play a protective role in the early stages of cellular stress [56]. Extensive mitochondrial networks found in *E. albidus* germ-line cysts prompted us to check if, in this case, hyperfusion could also be related to cellular stress. Therefore, in our third goal, we focused on reactive oxygen species (ROS). The ROS generate oxidative stress, and their overproduction, among other consequences, may lead to mitochondrial damage, as was shown in human cell lines [57]. In this context, the activity of mitochondria on the one hand, as well as their good quality on the other, is at odds in different tissues (both reproductive and others) [37, 58–60]. In *E. albidus* oogenesis, these processes must be implemented in a single compartment—the syncytial cysts—and seem to impact reproductive success significantly. Here, we analyzed the oxidative stress and antioxidant capacity in oogenesis and mature egg cells/oocytes. We compared them with nonreproductive somatic tissue.

## Materials and methods

### Animal material

Specimens of the white worm *E. albidus* (Henle, 1837) were bred under laboratory conditions in plastic boxes filled with potting soil. They were fed once a week with bread and vegetables soaked in water. For research purposes, only mature specimens with a well-visible clitellum were used.

### Fluorescence microscopy

To observe the general organization of the germ-line cysts, specimens of *E. albidus* were fixed in 4% formaldehyde (freshly prepared from paraformaldehyde) in phosphate buffered saline (PBS: NaCl, 137 mM; KCl, 2.7 mM; Na_2_HPO_4_ 8 mM;KH_2_PO_4_, 1.5 mM, pH 7.4) for 40 min at room temperature and washed in PBS. The OV, consisting of germ-line cysts in successive stages of oogenesis, were dissected, double stained with rhodamine-conjugated phalloidin (2 μg/mL; Sigma) to detect actin filaments and DAPI (4′,6-diamidino-2-phenylindole) (1 μg/mL) to counterstain the nuclei, for 40 min in darkness, washed in PBS and analyzed under an Olympus BX60 microscope equipped with the appropriate filters and an XC50 digital camera (Olympus, Tokyo, Japan) and the cellSens Standard software (Olympus, ver. 1.8.1).

To visualize the mitochondria and deoxyribonucleic acid (DNA) in living germ-line cysts JC-1 fluorochrome (5,5′,6,6′-tetrachloro-1,1′,3,3′-tetraethylbenzimidazolyl carbocyanine iodide) (T3168; Life Technologies, United States) and Hoechst 33342 (H1399; Life Technologies) were used. JC-1 is a cationic carbocyanine dye, accumulating in the mitochondria as monomers or aggregates, depending on the mitochondrial membrane potential (MMP) [61, 62]. When the MMP is low, JC-1 exists as a monomer and yields green fluorescence. In contrast, when the inner mitochondrial membrane is highly polarized, JC-1 monomers aggregate to form the so-called J-aggregates—these can be observed as red fluorescence. This means that all mitochondria generate the green signal, but only the active ones generate the red signal. We subtracted the red signal from green to calculate the percentage of inactive mitochondria. Dissected OV were rinsed in culture medium (Dulbecco’s phosphate buffered saline, DPBS, Sigma-Aldrich, St. Louis, MO, USA) and immersed in a solution with the JC-1 (1 mg/mL Dimethyl sulfoxide (DMSO)) (Sigma, Germany) in DPBS (5 μL JC-1/mL DPBS). As a positive control, carbonyl cyanide 3-chlorophenyl hydrazone (Sigma-Aldrich, St. Louis, MO, USA), a chemical inhibitor of oxidative phosphorylation, which depolarizes the electrochemical potential across the mitochondrial inner membrane, was used, according to the protocol of Sivandzade and others [63].

It should be noted that there are substantial limitations in the methods that can be used in *E. albidus*. Due to the complicated morphological arrangement of germ-line cysts (a group of 16 cells connected to the common cytoplasm via ring canals rich in cytoskeleton rims), we could not separate individual cells from cysts. Moreover, the cysts are covered by a somatic envelope because of which many alternative methods for labeling the mitochondria failed (e.g., active MnSOD, HSP70, and metallothionein antibodies, as well as MitoTracker Orange CMTMRos and DiOC6 live-cell stainings). As we were determined to link the ultrastructural observations of mitochondria with the cell functions, we chose those methods in which we could obtain valuable results. Even the JC-1 staining (explained later) was a challenge for us (as we also mentioned in [50]) due to the limited number of available germ-line cysts in the subsequent stages of oogenesis.

### Measurement of mitochondrial activity

Three-dimensional data sets of JC-1 staining were analyzed as volume-rendered data sets using Imaris (custom software developed by Bitplane Scientific Software, Zurich, Switzerland). For analysis, the components of germ-line cysts—entire young cysts with undifferentiated germ cells, nurse cells, and oocytes in differentiated cysts, as well as individual mature oocytes—were segmented using surface modeling that is available in Imaris using a manual outline. For each segmented part, the signals from JC-1 were detected using both 488 nm and 635 nm laser summarized channels and the spot detection Imaris module. An arbitrary mean intensity threshold for the summarized dye signal was used to detect the number of mitochondrial spots. As mitochondria spots, we defined the smallest detectable group of mitochondria. In all samples, the red channel (JC-1 activated) was used to determine the number of activated mitochondrial spots in a representative number of germ-line cysts. As a result, we calculated the mean percentage of activated mitochondrial spots in each cyst analysis compartment. Moreover, the Mann–Whitney test was used to assess statistical significance between cysts with growing oocytes and nurse cells with cytophore and compare germ and somatic cells in the level of active mitochondria. The obtained data were verified for occurrence of extreme values defined as follows: value > A + 2^*^C^*^(A − B) or value < B − 2^*^C^*^(A − B), where A is percentile 75%, B is percentile 25%, and C is the coefficient for outlier observations (1.5 is the usual value for extreme data). Results with *P* ≤ 0.05 were considered to be significant.

### LM, TEM, and SBEM

The dissected body segments with gonads were fixed in 2.5% glutaraldehyde in a 0.1 M phosphate buffer (pH 7.4) at room temperature for 1 h. Then the samples were washed in the same buffer and post-fixed with 3% potassium ferrocyanide in a 0.3 M cacodylate buffer mixed with an equal volume of a 4% aqueous solution of osmium tetroxide for 1 h. The samples were then washed three times for 5 min in ddH_2_O and incubated in a 1% solution of thiocarbohydrazide (Ted Pella) for 20 min at 60 °C. After that, they were washed three times for 5 min in ddH_2_O, placed in 2% aqueous osmium tetroxide for 30 min, and then rewashed three times for 5 min in ddH_2_O and incubated overnight in 1% aqueous uranyl acetate at 4 °C. The samples were then rinsed three times for 5 min in ddH_2_O, incubated in freshly prepared Walton’s lead aspartate [64] for 30 min at 60 °C, washed five times for 3 min in ddH_2_O, and dehydrated for 10 min in a series of 30, 50, 70, and 96% ethanol solutions, then placed in anhydrous 100% ethanol three times for 20 min, a 1:1 solution of acetone and ethanol for 15 min and twice for 15 min in 100% acetone. After dehydration, the samples were placed in a mixture of 50% Epoxy Embedding Medium (Sigma-Aldrich, St. Louis, MO, USA) in acetone for 3 h, then left overnight for acetone evaporation. The prepared material was embedded in Epoxy Embedding Medium between two layers of Aclar Electron Microscopy Sciences (EMS) and left to polymerize.

To analyze the material using LM, semithin sections (0.8-μm thick) were stained with methylene blue and analyzed using an Olympus BX60 microscope equipped with an XC50 digital camera (Olympus, Tokyo, Japan) and the cellSens Standard software (Olympus, ver. 1.8.1). To analyze the material using TEM, ultrathin sections (80 nm) were cut on a Leica Ultracut UCT ultramicrotome (Leica Microsystems, Wetzlar, Germany) or an RMC Boeckeler (RMC) Power XT ultramicrotome (RMC Boeckeler, Tucson, AZ, USA) and examined under a Hitachi H500 transmission electron microscope (Hitachi, Tokyo, Japan) at 75 kV.

**Three-dimensional reconstructions:** The samples were prepared in the same way as described above. After resin hardening, square samples, cut out with razor blades, were attached to aluminum pins (metal rivets, Oxford Instruments) with a very small amount of cyanoacrylate glue and then mounted to the ultramicrotome Ultracut UCT (Leica Microsystems, Wetzlar, Germany) and the block of the sample was trimmed. Next, samples were grounded with silver paint (Ted Pella, 16,062–15) to the pin and dried for 24 h. Stacks of images from serial 150 nm ultrathin sections were collected using a Sigma VP scanning electron microscope (Zeiss, Germany) equipped with an ultramicrotome chamber 3View2 (Gatan, United States) and Digital Micrograph software (Gatan, United States) and a back-scattered electron detector. The imaging parameters were: variable pressure 18 Pa, accelerating voltage 4 kV, aperture 15 μm, dwell time 7 μs, and pixel size 15 nm.

Three-dimensional reconstructions of the selected germ-line cysts at consecutive stages of oogenesis were based on a series of ultrathin sections. Microscopy Image Browser (MIB), a handy tool for image management, was used to prepare the three-dimensional models [65]. Objects of interest such as mitochondria, cell nuclei, ring canals, and cell membranes were segmented manually in MIB using a brush and threshold tool. The models obtained were visualized in a trial version of Amira (Thermo Scientific, Waltham, MA, USA).

### Measurement of mitochondria number, distribution, and morphology

Based on the 3D reconstructions, the mitochondria and mitochondrial clusters were segmented using the surface object detection function in Imaris (custom software developed by Bitplane Scientific Software, Zurich, Switzerland). The analyzed compartments (germ cells, cytophore, ring canals) were segmented using surface modeling, available in Imaris with a manual outline. In each detected mitochondrial cluster object, the number of mitochondria was calculated using an estimated fixed mitochondrion size. The mitochondrion size was determined by precisely manually contouring several single mitochondrion shapes on a series of ultrathin sections. The 3D objects were constructed using surface object detection, and for each object the volume estimate was calculated. The average volume value of 25 randomly selected objects was used to determine the final mitochondrion size. Using the calculated volume of the mitochondrial clusters and estimation of the single mitochondrion, the number of mitochondria in each cluster was calculated.

The level of mitochondrial connectivity was measured by confocal microscopy using surface modeling implemented in Imaris of all mitochondrial clusters. We estimated the volume of each mitochondrial cluster and calculated the number of standard-sized mitochondria fit in each cluster.

The 3D position coordinates of all the mitochondria detected inside the cytophore and germ cells in selected germ-line cysts were also measured. As a result, we calculated the Euclidean distances between each mitochondrion (detected using Imaris spot model) and the center of the ring canal in the cytophore and germ cells. The calculated distances were normalized by the maximal length of the cytophore or germ cells based on the localization of the mitochondria. Statistical analysis was performed using the R environment (ver. 3.4.2).

### Oxidative stress parameters

To analyze and compare the level of oxidative stress and the defense systems in germ-line cysts during oogenesis, we selected OV containing germ-line cysts, mature individual oocytes, and for comparison, the representative of somatic tissue—the body wall (BW). For this purpose, the levels of oxidative stress markers—catalase (CAT), glutathione S transferase (GST), total reduced glutathione (GSH)—and also the ABTS radical (total antioxidant capacity, TAC) were measured by calorimetric methods using a UV–vis spectrometer (TECAN Infinite M200, Austria). The homogenates used for analysis were prepared as follows: the BW, OV, and oocytes were collected and placed in Eppendorf tubes in 100 μL of a 0.1 M Sørensen buffer (pH 7.4). Next, every sample was homogenized and centrifuged (15 000 × g, 10 min, 4 °C). The submitochondrial fraction was collected and stored at −70 °C. Before analyses, the total protein concentration in every sample was determined according to Bradford [66] using bovine serum albumin (BSA, protein content >95%, Fluka) as the standard. Moreover, the generation of (ROS + cells) was measured by MUSE® cell analyzer (Millipore, Billerica, MA, USA) flow cytometer. Dissected tissues were placed in 0.1 M PBS (pH 7.4) and gently homogenized in a homogenizer (Minilys®, Bertin Technologies) to obtain the cell suspension. Measurements were taken using the Muse® oxidative stress kit according to the manufacturer’s recommendations.

CAT [EC 1.11.1.6] was measured according to Orr [67]. A mixture of CAT supernatant (1 μL) with 0.05 M phosphate Sørensen buffer pH 7.4 (99 μL) and 10 mM hydrogen peroxide (50 μL) was prepared. The change in absorbance was immediately measured at 230 nm for 40 s (with an 8-s interval).

GST [EC 2.5.1.18] activities were determined spectrophotometrically as described by Yu [68], using CDNB (1-chloro-2,4,dinitrobenzene) as the substrate. The reaction medium with 1 mM of reduced GSH solution in 0.1-M phosphate Sørensen buffer pH 7.4 (296 μL) and supernatant (2 μL) was incubated at room temperature for 1 min. Then, 1.0-mM CDNB solution in absolute ethanol (2 μL) was added to the samples. Enzyme activity was determined by continuously monitoring the change in absorbance at 340 nm for 5 min (with a 1-min interval).

GSH level was measured according to the method described by Griffith [69]. For this purpose, 5% trichloric acid (0.1 mL) was added to the supernatant (0.1 mL), then centrifuged at 1000 × g for 10 min at room temperature. The supernatant was neutralized with 125-mM sodium phosphate buffer pH 7.45 containing 6.3 mM Ethylenediaminetetraacetic acid (EDTA) (0.8 mL). For further analyses, solutions of glutathione reductase (58-μL GR in 30-mL H_2_O_2_), 5,5-dithiobis (2-nitrobenzoic) acid (23.7 mg DTNB (5,5-dithiobis (2-nitrobenzoic) acid) in 10 mL of phosphate Sørensen buffer), and reduced nicotinamide adenine dinucleotide phosphate (7.5-mg NADPH in 30 mL phosphate Sørensen buffer) were prepared. The reaction mixture contained 26 μL of supernatant, 107 μL of GR, 26 μL of DTNB, and 90.7 μL of NADPH. Glutathione absorbance was recorded every minute for 5 min at 412 nm. The obtained results were compared with a standard curve prepared for a series of GSH dilutions starting from 2000-μM GSH.

TAC assay was performed according to Re and coauthors [70]. The radical of 2,20-azino-bis(3-ethylbenzthiazoline-6-sulphonic acid) (ABTS) was prepared by reacting ABTS (19.5 mg) with potassium persulfate (3.3 mg) in a 0.1 M sodium phosphate buffer with pH 7.4 (7 mL) for 12–16 h in darkness. The blue-green solution was maintained at −20 °C. Before use, ABTS was diluted with water. The reaction mixture contained 10 μL of supernatant and 90 μL of diluted ABTS. Decolorization of ABTS was measured at 415 nm. The results were compared with a standard curve prepared for a series of 6-hydroxy-2,5,7,8-tetramethylchroman-2- carboxylic acid (Trolox) dilutions starting from 250-μM Trolox. The results are reported as mean values ± SD. Normality was checked with the Kolmogorov–Smirnov test. The data were tested for the homogeneity of variance using Levene’s test of the equality of error variances. Tukey’s multiple comparisons test with a post hoc one-way analysis of variance was used to determine differences between the experimental groups: the OV containing germ-line cysts, mature individual oocytes, and the BW of *E. albidus*. Results with *P* ≤ 0.05 were considered to be significant. The data were analyzed using GraphPad Prism ver. 6.

**Figure 2:**
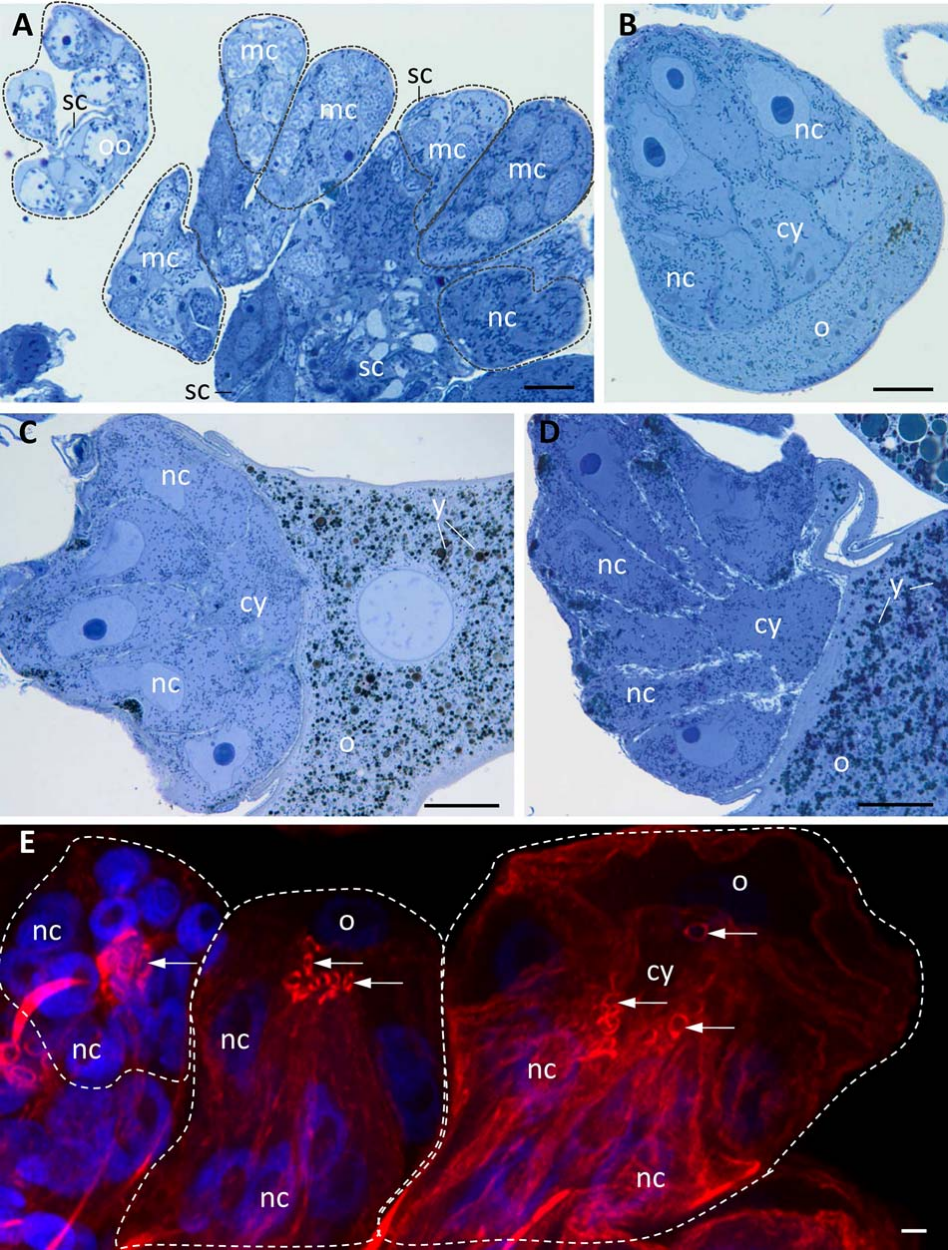
General organization of germ-line cysts in *E. albidus*. (A) Ovary fragment with several germ-line cysts (*encircled*). Within each cyst, germ cells at the same stage of oogenesis are visible: oogonia (*oo*); meiotic cells (*mc*), in the first prophase of meiosis; and nurse cells (*nc*). Germ-line cysts are accompanied by flattened somatic cells (*sc*). (B–D) Germ-line cysts during advanced oogenesis (B, previtellogenesis; C–D, vitellogenesis) containing nurse cells (*nc*) and growing oocytes (*o*) with gathering nutritive material, yolk (*y*). Note the cytophore (*cy*). (E) Three germ-line cysts containing nurse cells (*nc*) and oocytes (*o*) with F-actin labeled by rhodamine-conjugated phalloidin (red signal). F-actin is enriched in rims of ring canals (*arrows*) that connect each germ cell (*nc*, nurse cells; *o*, oocyte) to the central cytophore (*cy*). DAPI counterstained cell nuclei (blue signal). (A–D) Light microscopy, semithin sections stained with methylene blue; (E) fluorescence microscopy, merged images were taken using the laser 405 nm and DAPI filter—blue fluorescence and laser 635 nm phycoerythrin (PE) Texas Red filter—red fluorescence; scale = 10 μm.

## Results

### Germ-line cyst architecture during oogenesis

The fully formed germ-line cysts of *E. albidus* were described recently [23, 50] and in the present study. They consisted of 16 germ cells connected with the common mass of cytoplasm, termed the cytophore (Figures 1B and 2). The cytophore was located in the cyst center, and as a rule, each cell was connected to it via a wide cytoplasmic channel (from 3.7to 5.5 μm [23], the so-called ring canal (Figure 2E). The rims of ring canals were lined with F-actin forming characteristic ring-like accumulations (Figure 2E). Ovaries found in mature specimens (well-developed clitellum) were composed of a dozen or so cysts at successive stages of oogenesis (Figure 2A). It should be noted here that germ-line cysts were accompanied by flattened somatic cells (Figure 2A and [23, 51, 71]).

**Figure 3:**
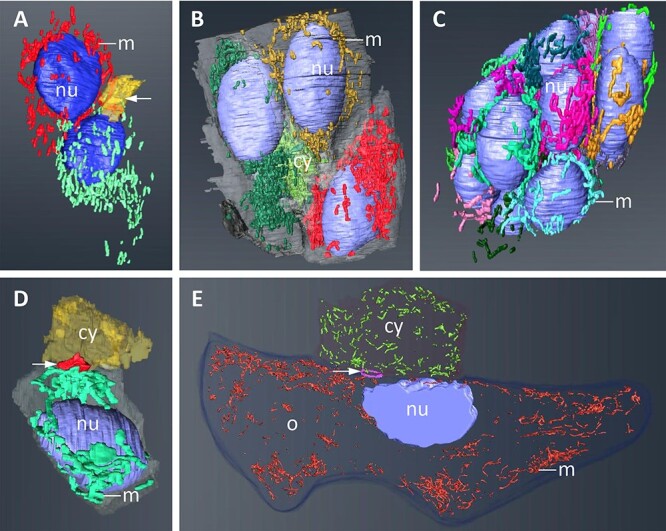
Snapshots from 3D reconstructions of germ-line cysts at subsequent stages of oogenesis. All reconstructions are based on serial ultra-thin sections obtained by SBEM technique. Within cysts, reconstructed mitochondria (*m*) belonging to a given cell are marked in one color. (A) Two-celled cyst, connecting via broad and irregular ring canal (*single short arrow*), (B) 4-celled cyst with three cells and the cytophore (*cy*) visible, (C) 16-celled cyst with small cytophore (*cy*) inside, (D) one cell from the 16-celled cyst with ring canal (*single short arrow*) connecting it to the cytophore (*cy*), and (E) part of the growing oocyte (*o*), its ring canal (*single short arrow*) and the cytophore (*cy*). Germ cell nuclei (*nu*), mitochondria (*m*).

**Figure 4:**
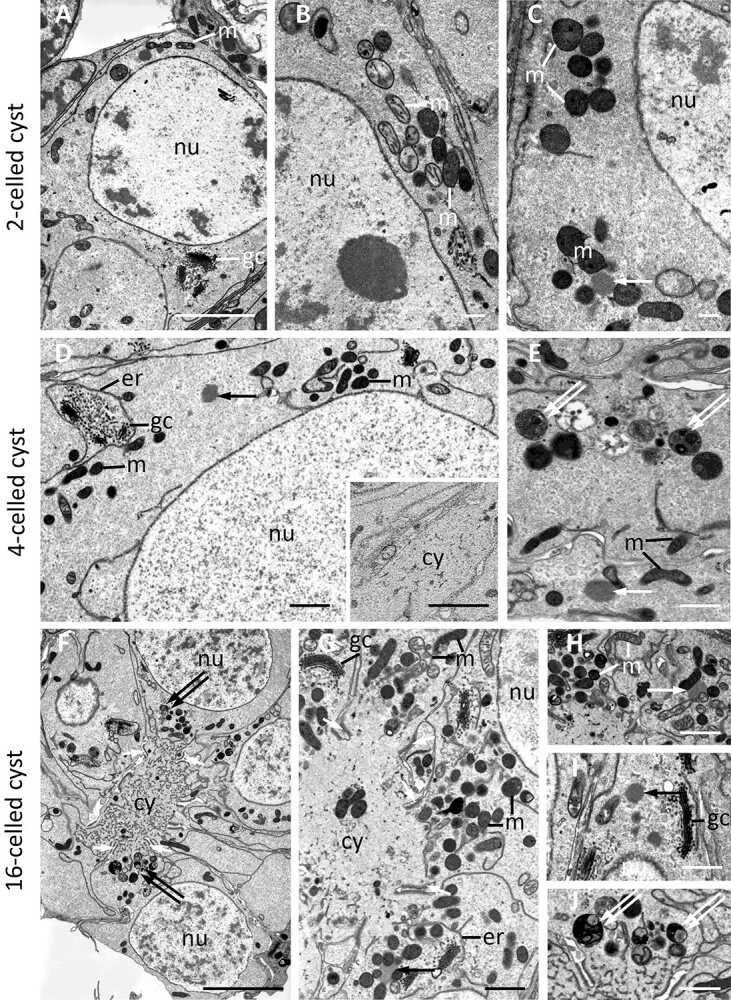
Ultrastructural details of germ-line cysts at consecutive stages of oogenesis: (A–C) 2-celled cysts consisting of oogonia, (D and E) cystocytes from 4-celled cysts, and (F–J) 16-celled cysts with cystocytes in the first prophase of meiosis. Note numerous profiles of coexisting mitochondria (*m*) with electron-light and high electron-dense matrix; cell nuclei (*nu*); patches of electron-dense granulo-fibrillar nuage material (*single arrow*). Golgi complexes (*gc*), usually enclosed by tubules of endoplasmic reticulum (*er*); autophagosomes containing remnants of cell organelles (*double arrows*); note ring canals, connecting cystocytes to the cytophore (*cy*), that is, between the two ring canal rims (*single short arrow*). Note the cytoplasm of the cytophore, filled with thin tubules and devoid of cell organelles, except rare mitochondria. Transmission electron microscopy, ultrathin sections, scale = 1 μm.

**Figure 5:**
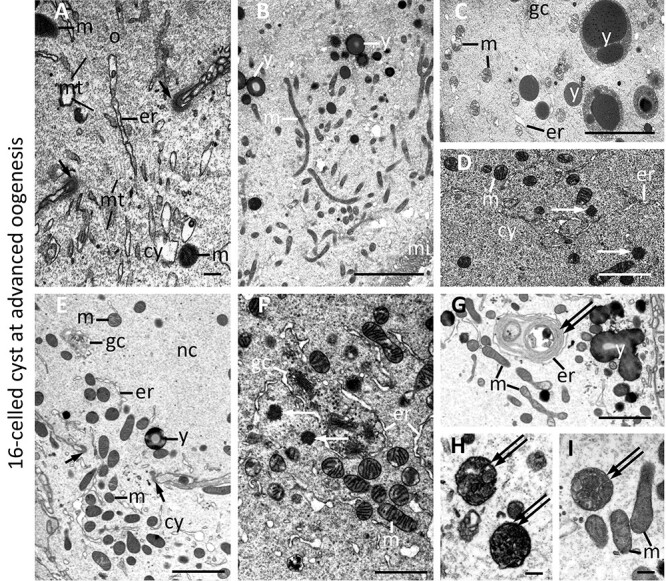
Ultrastructure of germ-line cysts at advanced oogenesis. (A) ring canal (with clearly visible ring canal rim—*single short arrow*) connecting previtellogenic oocyte (*o*) with cytophore (*cy*), (B) early vitellogenic oocyte, (C) more advanced vitellogenic oocyte, (D) cytophore cytoplasm, (E) part of nurse cell (*nc*) connected via the ring canal (*single short arrow*) with the cytophore (*cy*), (F–I) details of nurse cells that accompany large vitellogenic oocyte. Note numerous mitochondria profiles (*m*) in the cytoplasm of germ cells and cytophore and within ring canals; ring canal rim (*short arrows*); microtubule (*mt*); Golgi complexes (*gc*); endoplasmic reticulum (*er*); nutritive material—yolk (*y*); nuage material (single *arrow*); autophagosomes with remnants of cell organelles (*double arrows*); microvilli on the oocyte surface (*mi*). Transmission electron microscopy, ultrathin sections, scale = 1 μm.

In the present study, we concentrated on early cyst generations, that is, on cysts consisting of 2, 4, and 16 morphologically identical interconnected cells (termed cystocytes) as well as on fully differentiated cysts with one oocyte and 15 nurse cells (Figures 1B, 2, and 3). Microscopic analysis revealed that the interconnected cells were initially in the same phase of the cell cycle, and there was synchronous development of cystocytes in a given cyst (Figure 2A). The formation of the cytophore inside was simultaneously correlated with the cyst development. In the youngest 2-celled cyst, the ring canal interconnecting both cystocytes was thin and elongated, and there was no noticeable cytophore (Figure 3). Later on, in 4-celled cysts between the four ring canals, a small portion of common and anuclear cytoplasm was clearly visible (Figure 3B). This cytoplasm mass was an initial cytophore. At the ultrastructural level, the cytoplasm of the initial cytophore and within ring canals differed from the cytoplasm of cystocytes, that is, it was granular and more electron lucent and was devoid of cell organelles (Figure 4D inset; see also in [23]). In the 16-celled cyst (after the last round of divisions), the cytophore was a small, spherical cytoplasmic mass but irregular in outline, located just within the cyst center. It was still an initial cytophore, filled with granular electron-lucent cytoplasm poor in cell organelles but rich in endoplasmic reticulum (ER) cisternae (Figures 3 and 4F and G). In cysts where cystocytes were already differentiated into an oocyte and nurse cells, the cytophore was more voluminous, and its cytoplasm was morphologically identical to that filling germ cells and was filled with numerous cell organelles such as mitochondria and ER cisternae (Figures 2B–D, 3, and 5).

The synchronous development of all interconnected cells lasted until early meiosis (at least till pachytene); in all cells, the synaptonemal complexes were formed (Figure 1E in [23]). Then, one cell (oocyte) continued the meiosis, whereas the 15 remaining cells became nurse cells. The oocyte became enlarged considerably, gathering the cell organelles and absorbing yolk precursors by intensive endocytosis and authosynthesis [51, 71] and became the future egg cell (Figures 1B, 2B–, 3, and 5A–F). Morphological observations of cell organelles (as mitochondria and ER) present within ring canals and the cytophore (Figure 5A and E; Figure 3 in [71]; Figure 3D in [23]) suggested that nurse cells may provide the oocyte with these cell components. Nurse cells did not grow considerably and gather nutrients; at the end of oogenesis, they were most probably absorbed by the oocyte [23, 51].

### Mitochondrial ultrastructure

SBEM methodology used for the analysis of ultrastructure strongly marks cellular membranes; therefore, such structures as mitochondrial cristae, ER cisterns, and Golgi complexes are strongly and clearly visible. Interestingly, mitochondria found in all analyzed cyst compartments (cystocytes, nurse cells, oocytes, cytophore) could be classified into two categories regarding the electron density of the matrix: electron light versus high electron density matrix (Figures 4 and 5). These two kinds of mitochondria possessed cristae (hard to visible in mitochondria with electron dense matrix) and coexisted in the same cytoplasm portion. No regularities in their distribution in the cells and cytophore were observed (Figures 4 and 5).

Similarly, in the cystocyte cytoplasm, nurse cells, oocytes, and also in the cytophore of the cyst containing nurse cells and the oocyte, small patches of medium electron-dense material closely associated with mitochondria (often in direct contact with them) were usually observed (Figures 4C–E and G–I, and 5D–F). Such an unbounded granulo-fibrillar material is characteristic of the germ cells of numerous animals and is collectively termed nuage [72, 73]. Together with numerous mitochondria and small nuage accumulations in the cystocyte cytoplasm, long tubules of the ER and numerous Golgi complexes were also observed (Figures 4A, C, G, and I, and 5A and C–G).

In cystocytes forming 4-celled and 16-celled cysts, and nurse cells coming from more advanced cysts, autophagosomes (autophagy vesicles) were found between typical organelles. Autophagosomes contained remnants of organelles such as mitochondria and Golgi complexes (Figures 4E, F, and J, and 5H and I). It is worth mentioning that in nurse cells accompanying the vitellogenic oocyte, organellar remnants enclosed within autophagosomes, tightly surrounded by ER cisternae, were noted (Figure 5G).

### Mitochondrial morphology and distribution

**Figure 6:**
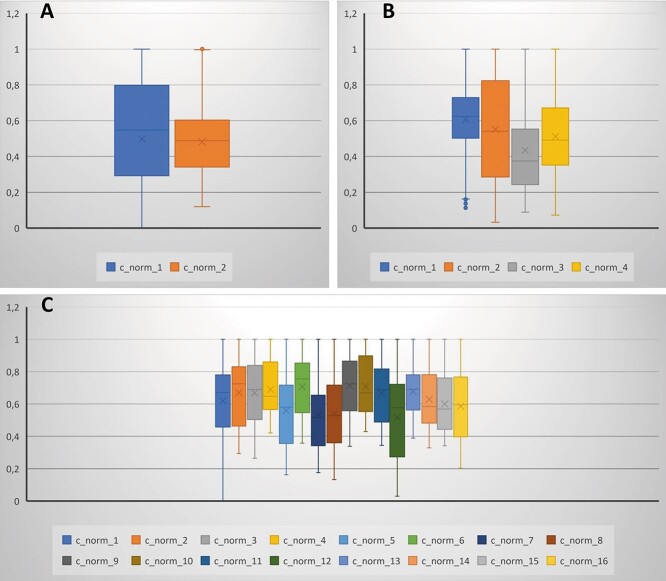
Schematic diagram of mitochondria distribution in cells within the particular cysts [2-celled cyst in (A), 4-celled cyst in (B), and 16-celled cyst in (C)], visible as normalized distances of mitochondria from the ring canals. Each bar relates to the mitochondria from a single cell in a cyst. Point 0 refers to the part of the cell where the ring canal is located, while point 1 means the most distant end of the cell. As cells have different shapes and sizes, the normalized distance was used. Diagrams of a particular cell are marked as example: c_norm_1, where 1 means the number of a cell within the cyst.

The use of such microscopic methods as SBEM, TEM, and LM generated data which revealed the morphology and distribution of mitochondria in all compartments of germ-line cysts, at successive stages of oogenesis. Moreover, we reconstructed in 3D at the ultrastructural level mitochondria in cystocytes, cytophores, and ring canals of 2-, 4-, and 16-celled cysts, and in a part of a cyst with growing oocyte and nurse cells (Figure 3, Supplementary Files S1–S5). To analyze changes in the morphology and organization of mitochondria within germ-line cysts, quantitative data were obtained: (1) the number of mitochondria in the particular cyst compartments and the mitochondria density (Table 1); (2) the volume of an individual mitochondrion and the volume of mitochondria fused into larger clusters, interpreted as mitochondrial networks, and classified in appropriate groups, and calculated percentage of the appropriate cluster, as shown in detail in Table 2 (selected exemplary diagrams from these analyses, including the level of mitochondrial connections are shown in Supplementary File S6); and (3) an analysis of the distribution of mitochondria in the particular cysts’ cells, based on calculated normalized distances of mitochondria from the ring canals, presented as a schematic diagram in Figure 6. The mitochondria in germ-line cysts were measured and the mean volume of a single mitochondrion was 0.0235 ± 0.01 μm (SD), *n* = 25.

**Table 1:** Measurements of mitochondrial density—number of mitochondria per unit of cytoplasm volume, in selected compartments of germ-line cysts.

	Cell no.	Cell volume	Volume of nucleus	Volume of cytoplasm	Number of mitochondria	Number of mitochondria per unit of cytoplasm volume
2-celled cyst	1	737	254	483	1382	2.86
	2	808	260	548	1720	3.14
						**mean value = 3.00**
4-celled cyst	1	887	271	616	2007	3.26
	2	643	224	419	1136	2.71
	3	667	215	452	1711	3.79
	4	765	256	509	2117	4.16
						**mean value = 3.48**
16-celled cyst	1	320	145	175	670	3.83
	2	300	135	165	839	5.08
	3	312	135	177	882	4.98
	4	323	143	180	646	3.59
	5	297	135	162	521	3.22
	6	332	131	201	594	2.96
	7	318	131	187	581	3.11
	8	318	139	179	752	4.20
	9	319	129	190	711	3.74
	10	291	123	168	686	4.08
	11	342	147	195	645	3.31
	12	291	131	160	627	3.92
	13	328	144	184	579	3.15
	14	310	135	175	626	3.58
	15	312	133	179	579	3.23
	16	283	135	148	686	4.64
						**mean value = 3.79**
Cyst with growing oocyte	oocyte	4413	530	3883	11 623	2.99
	cytophore	955	0	955	2045	2.14

**Table 2:** Measurements of mitochondrial morphology—the number of counts (NoC), the volume (Vol), and the percentage (%) of single mitochondria (range of value 0–1) and different clusters of mitochondrial networks (range of value 2–10, 11–100, 101–1000, and 1001–10 000) in the germ cells (C1–C16) in 2-celled, 4-celled, and 16-celled cysts.

2-celled cyst	Range of Value	NoC C1	Vol (μm)	%	NoC C2	Vol (μm)	%						
	0–1	7	0.129074	0.4	17	0.27	0.6						
	2–10	132	14.35194	39.0	115	12.99	29.9						
	11–100	38	19.38418	52.7	47	25.01	57.5						
	101–1000	1	2.90854	7.9	2	5.22	12.0						
	1001–10 000	0	0	0	0	0	0						
4-celled cyst	**Range of Value**	**NoC C1**	**Vol (μm)**	**%**	**NoC C2**	**Vol (μm)**	**%**	**NoC C3**	**Vol (μm)**	**%**	**NoC C4**	**Vol (μm)**	**%**
	0–1	22	0.25609	0.5	17	0.24	0.8	29	0.35	0.7	17	0.23	0.4
	2–10	87	9.797612	18.5	91	8.71	28.7	73	6.06	12.1	107	10.29	17.0
	11–100	30	14.56466	27.5	24	13.41	44.2	11	6.01	11.9	19	9.74	16.1
	101–1000	3	28.41271	53.6	2	7.97	26.3	2	13.18	26.2	4	40.24	66.5
	1001–10 000	0	0	0	0	0	0	1	24.67	49.1	0	0	0
16-celled cyst	**Range of Value**	**NoC C1**	**Vol (μm)**	**%**	**NoC C2**	**Vol (μm)**	**%**	**NoC C3**	**Vol (μm)**	**%**	**NoC C4**	**Vol (μm)**	**%**
	0–1	2	0.038629	0.2	2	0.02	0.1	0	0.00	0.0	1	0.01	0.1
	2–10	8	1.180097	6.3	7	0.68	2.9	12	1.33	4.6	11	1.22	6.0
	11–100	11	5.705579	30.6	1	0.29	1.2	9	3.48	11.9	7	3.88	19.2
	101–1000	2	11.70723	62.8	2	22.67	95.8	0	0.00	0.0	1	15.14	74.8
	1001–10 000	0	0	0	0	0	0	1	24.35	83.5	0	0	0
	**Range of Value**	**NoC C5**	**Vol (μm)**	**%**	**NoC C6**	**Vol (μm)**	**%**	**NoC C7**	**Vol (μm)**	**%**	**NoC C8**	**Vol (μm)**	**%**
	0–1	1	0.018753	0.1	4	0.08	0.5	3	0.06	0.3	5	0.08	0.4
	2–10	14	1.908437	12.4	20	2.23	13.3	18	2.28	13.8	21	1.68	8.2
	11–100	19	13.52435	87.5	14	10.19	61.0	13	6.48	39.2	8	5.21	25.5
	101–1000	0	0	0	1	4.20	25.2	1	7.70	46.7	2	13.48	65.9
	1001–10 000	0	0	0	0	0	0	0	0	0	0	0	0
	**Range of Value**	**NoC C9**	**Vol (μm)**	**%**	**NoC C10**	**Vol (μm)**	**%**	**NoC C11**	**Vol (μm)**	**%**	**NoC C12**	**Vol (μm)**	**%**
	0–1	4	0.048112	0.2	4	0.05	0.3	6	0.09	0.5	1	0.02	0.1
	2–10	14	1.605843	8.1	9	0.53	3.0	26	2.65	14.3	23	2.16	12.7
	11–100	14	10.16665	51.0	9	5.93	33.8	14	9.69	52.3	14	7.58	44.7
	101–1000	1	8.11587	40.7	3	11.06	62.9	1	6.11	33.0	2	7.19	42.4
	1001–10 000	0	0	0	0	0	0	0	0	0	0	0	0
	**Range of Value**	**NoC C13**	**Vol (μm)**	**%**	**NoC C14**	**Vol (μm)**	**%**	**NoC C15**	**Vol (μm)**	**%**	**NoC C16**	**Vol (μm)**	**%**
	0–1	4	0.076552	0.5	9	0.10	0.6	1	0.02	0.1	5	0.08	0.4
	2–10	17	1.064155	6.5	7	0.76	4.5	23	2.41	15.6	21	1.68	8.2
	11–100	6	4.170327	25.5	13	7.87	46.6	15	8.63	55.9	8	5.21	25.5
	101–1000	2	11.05905	67.6	1	8.16	48.3	1	4.38	28.4	2	13.48	65.9
	1001–10 000	0	0	0	0	0	0	0	0	0	0	0	0

#### Mitochondria in the 2-celled cyst

In the 3D visualized 2-celled cyst, mitochondria were distributed almost evenly in the cell cytoplasm, scattered around the cell nuclei (Figure 3; Supplementary File S1). The characteristic feature was the lack of mitochondria in the cytoplasm adjoining the ring canal and within the ring canal itself (Figures 3 and 6; Supplementary File S1). Using the estimated volume of a single mitochondrion, the density of mitochondria was about 3.0 per unit of cytoplasm volume (Table 1). Moreover, the individual mitochondria in cystocytes numbered 7 in the first and 17 in the second cell, representing, respectively, 0.4 and 0.6% of the total cystocyte mitochondrial volume (Table 2). To assess the level of mitochondrial connectivity, we measured how many of such individual mitochondria were included in the larger, interconnected ones (clusters, mitochondrial networks). The analysis showed that about 34% of the total mitochondrial volume comprised larger clusters corresponding (by volume) to 2–10 individual ones, whereas 55% of the volume comprised mitochondrial networks corresponding to 11–100 individual mitochondria (Table 2). The largest mitochondrial networks noted in 2-celled cysts corresponded to 101–1000 individual mitochondria, and that was about 10% of the total mitochondrial volume (Table 2).

#### Mitochondria in the 4-celled cyst

The morphology and distribution of mitochondria in a 4-celled cyst was different. In each cell, mitochondria formed two aggregates, located at two cell poles—one near the ring canal and the second on the opposite cell pole, thus in the proximal and distal cell portions respectively (Figures 3 and 6; Supplementary File S2). However, in the close proximity of ring canals and within the cytoplasm of the initial cytophore mitochondria, were not found (Figures 3 and 6; Supplementary File S2). The calculation of the mitochondrial volume showed that in one cell there was higher mitochondrial density than in the others, and it reached the value of 4.16 (number of single mitochondria per unit of cytoplasm volume; Table 1). The measurement of the number of individual mitochondria and larger clusters in cystocytes showed approximately 21 single mitochondria. It represented approximately 0.6% of the total cystocyte mitochondrial volume (Table 2). The analysis of the level of mitochondrial connection showed larger clusters consisting of 2–1000 individual ones, in the case of three cystocytes. Additionally, a higher percentage of the total mitochondrial volume was found in the case of the larger networks (i.e., 11–100 and 101–1000) than 2–10 (Table 2). In contrast, in one cell, besides individual mitochondria and the networks described above (i.e., 2–10, 11–100, and 101–1000), it was revealed that one much more extensive network corresponding to up to 10 000 individual mitochondria was also formed, and that was about 49% of the total mitochondrial volume of this cell (Table 2).

#### Mitochondria in the 16-celled cyst

In the 16-celled cyst, in which all cystocytes were morphologically undifferentiated, and all of them were in the first prophase of meiosis (their nuclei were characteristic for this phase of synaptonemal complexes), mitochondria were elongated, branched, and scattered in the cytoplasm (Figure 3, Supplementary Files S3 and S4). Accumulations of mitochondria were also distributed in the vicinity of ring canals; however, in general, mitochondria were located mainly at some distance from the ring canal and occupied a central position around the cell nuclei (Figure 6). Additionally, ultrastructural analysis of such cysts revealed that a few scattered mitochondria could also be found in the cytoplasm of the cytophore (Figure 4F and G). The measurement of cell volume showed that cystocytes were on average 2.5 times smaller than cells in the 4-celled cyst, and the number of mitochondria was significantly smaller (from 521 to 839). However, the number of mitochondria per unit of cytoplasm volume reached the mean value of 3.79, which indicates that in the 16-celled cyst, the density of mitochondria was even a little higher compared to the 4-celled cyst (Table 1). Moreover, in these cystocytes, there were only a few (from 0 to 9) individual mitochondria, and they represented about 0.3% of the total cystocyte mitochondrial volume. The analysis of the level of mitochondrial connection showed that in cystocytes, there were several clusters corresponding to 2–100 individual ones. In 14 cystocytes, there were 1–3 larger mitochondrial networks corresponding to 101–1000 individual mitochondria. What is worth noting, in one cystocyte, a single extensive mitochondrial network was noted. It corresponded to 1001–10 000 individual mitochondria and represented as much as 83.5% of the total mitochondrial volume (Table 2).

#### Mitochondria in the 16-celled cyst at advanced oogenesis

In cysts at advanced oogenesis, connecting cells already differentiated into the oocyte and nurse cells, mitochondria were numerous, and analyses of nurse cells in LM and TEM showed abundant accumulations of these organelles (Figure 2B–D). Numerous mitochondria were also observed within the ring canals and in the cytophore (Figure 5A, D, and E). Together with a bundle of microtubules, they looked like they were passing through the ring canals (Figure 5A). Although in the present study we did not reconstruct in 3D (and thus did not make quantitative analysis) mitochondria in nurse cells, our observations described above fully agree with a recent study, which was devoted to the 3D analysis of mitochondria in nurse cells of *E. albidus* [50]. Here we prepared the 3D reconstruction of mitochondria in a part of the growing (vitellogenic) oocyte, its ring canal, and cytophore. As the oocyte was too large to reconstruct it as a whole, and our goal was to measure the mitochondria and determine the level of their connection, we performed the reconstruction based on several dozen sections from a cell fragment near its connection with the cytophore. Abundant mitochondria scattered between the nutritive material accumulating in the ooplasm and passing through the ring canal were found. Long, branched mitochondria in the ooplasm were also clearly visible during the analysis of growing oocytes in TEM (Figure 5A–C; Supplementary File S5).

The measurement of mitochondria revealed a higher density of mitochondria in the ooplasm than in the cytophore (2.99 vs. 2.14) (Table 1). What is interesting, both in the oocyte and cytophore, is that the number of individual mitochondria was relatively high (280 and 86, respectively), and they represented 1.9% (in the oocyte) and 2.4% (in the cytophore) of the total mitochondrial volume. Similar to early germ-line cysts described above, mitochondria formed more extensive networks. The analysis of mitochondrial connection-level showed that the mitochondrial clusters with 11–100 individual mitochondria represented the highest percentage of the total mitochondrial volume (42.4% in the oocyte and 51.5% in the case of the cytophore). The maximal range of fused mitochondria was 101–1000 individual mitochondria in a single mitochondrial network. In the cytophore, only one such cluster was noted (7.3% of the total mitochondrial volume), whereas in the oocyte, nine such clusters were identified (30% of the total mitochondrial volume) (Table 2).

### Changes in mitochondrial activity in oogenesis

**Figure 7:**
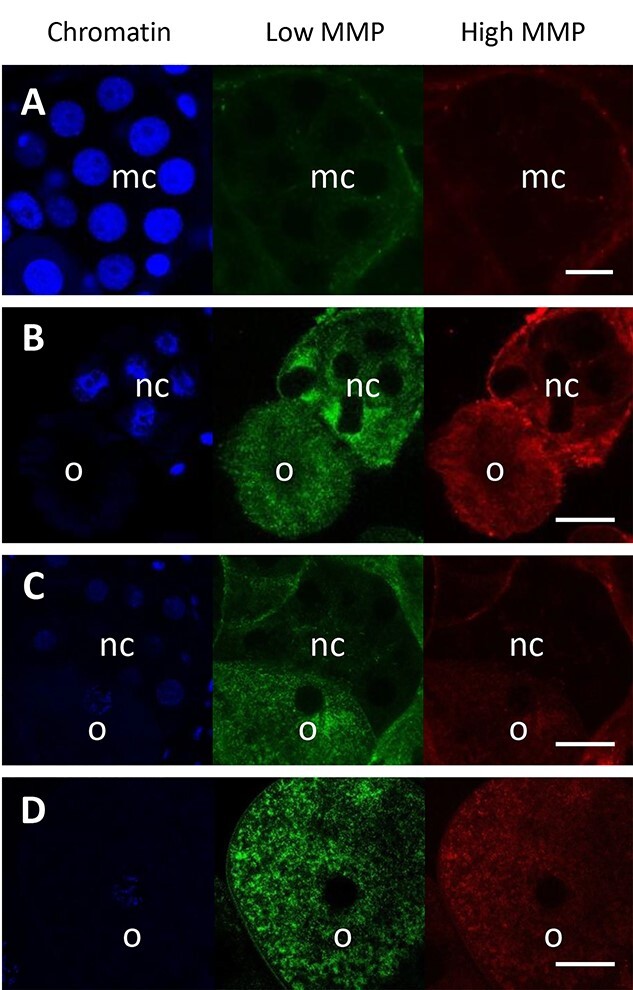
Single focal planes of germ-line cysts stained with JC-1 to visualize mitochondria with low MMP (low MMP—green), mitochondria with high MMP (high MMP—red), and Hoechst 33342 to counterstain the chromatin in cell nuclei (blue). (A) The 16-celled germ-line cyst with undifferentiated meiotic cystocytes (*mc*). (B) The germ-line cyst with nurse cells (*nc*) and young oocyte (*o*). (C) The germ-line cyst with nurse cells (*nc*) and large oocyte (*o*). (D) The oocyte (*o*). Fluorescent confocal microscopy, whole-mounted preparation, images in the first column (blue fluorescence) were taken using the laser 405 nm and DAPI filter, in the second column (green fluorescence) using the laser 488 nm and FITC filter, and in the third column (red fluorescence) with the use of the laser 635 nm and PE Texas Red filter, scale = 50 μm.

The occurrence of extensive mitochondrial networks in all analyzed cysts prompted us to conduct parallel studies of mitochondrial function. One probable function of connected mitochondria is more efficient ATP production (see Introduction); thus, we decided to analyze mitochondrial activity and its changes at subsequent stages of cyst development. We used JC-1 staining, which visualizes low MMP and high MMP as green and red fluorescence signals, respectively (Figure 7). As all mitochondria generate the green signal, but only the active ones generate the red signal, we assessed the percentage of active mitochondria characterized by high membrane potential by subtracting the red signal from green. We analyzed successive stages of oogenesis: (1) early cysts composed of undifferentiated cystocytes; (2) cysts at advanced oogenesis (with oocytes and nurse cells) as well as in (3) large vitellogenic oocytes; and also in (4) somatic cells that surrounded the cysts and formed their thin envelope (Table 3; Figure 7).

**Table 3:** Ratio of active and inactive mitochondria in selected compartments of germ-line cysts presented as a percentage of mitochondria with low (% of inactive mitochondria) and high (% of active mitochondria) membrane potential.

Selected components	No.	% of active mitochondria		% of inactive mitochondria	
Young cysts with	1	32.2		67.8	
undifferentiated cystocytes	2	30.4		69.6	
	3	28.5		71.7	
	4	49.8		50.2	
	5	56.3		43.7	
	6	55.0		45.0	
	7	56.0		44.0	
	8	38.6		61.4	
	**Average**	**43.3**		**56.7**	
Cysts with growing oocytes vs. nurse cells +cytophore		**Oocyte**	**Nurse cells + Cytophore**	**Oocyte**	**Nurse cells + Cytophore**
	1	47.8	62.0	52.2	38.0
	2	42.8	41.6	57.2	58.4
	3	47.1	52.2	52.9	47.8
	4	52.5	57.0	47.5	43.0
	5	56.7	61.3	43.3	38.7
	6	52.4	55.0	47.6	45.0
	7	51.4	54.5	48.6	45.5
	8	51.0	54.2	49.0	45.8
	9	50.1	52.2	49.9	47.8
	**Average**	**50.2**	**54.4**	**49.8**	**45.6**
Large vitellogenic oocytes	1	57.7		42.3	
	2	59.8		40.2	
	3	19.6		80.4	
	**Average**	**45.7**		**54.3**	
Somatic cells	1	59.8		40.2	
	2	64.0		36.0	
	3	59.8		40.2	
	4	55.7		44.3	
	**Average**	**59.8**		**40.2**	

The analysis of the mitochondrial spots in the selected cyst compartments showed on average about (1) 43% active mitochondria versus 57% inactive ones in early germ-line cysts; (2) 50% active and 50% inactive mitochondrial spots in growing oocytes, whereas in the same cysts, the percentage of mitochondrial spots in cyst compartments including nurse cells and the cytophore was 54.4% for active and 45.5% for inactive mitochondria; (3) 46% of active mitochondrial spots and 54% of inactive ones in large and individual vitellogenic oocytes; and (4) 60% active versus 40% inactive mitochondria in somatic cells. The detailed measurements and the percentages of mitochondrial spots with high and low membrane potential within particular selected compartments are shown in Table 3. These analyses indicated that there were both active and inactive mitochondria in germ-line cysts and somatic cells simultaneously. There were no significant discrepancies in the relative percentages of both energy states. The largest difference (about 20% more of active mitochondria) was noted in somatic cells, and these cells showed, at the same time, the statistically significant differences of active mitochondria among all analyzed compartments, and hence concerning the germ-line (*P* = 0.0093). Simultaneously, there was a lower percentage of active mitochondria among the germ-line cysts. However, statistically significant differences were noted between nurse cells and the cytophore, in which the ratio of active mitochondria was higher than in growing oocytes within the same cyst (*P* = 0.0474). When we analyzed large individual oocytes (we were only able to study three of these cells), we found apparent differences between cells. In two of them, the active mitochondria were slightly more numerous than the inactive ones. In contrast, in the third oocyte, the inactive mitochondria had a significant predominance (80.4 vs. 19.6%, see Table 3).

### Analysis of the level of oxidative stress and the antioxidant cellular defense system

**Figure 8:**
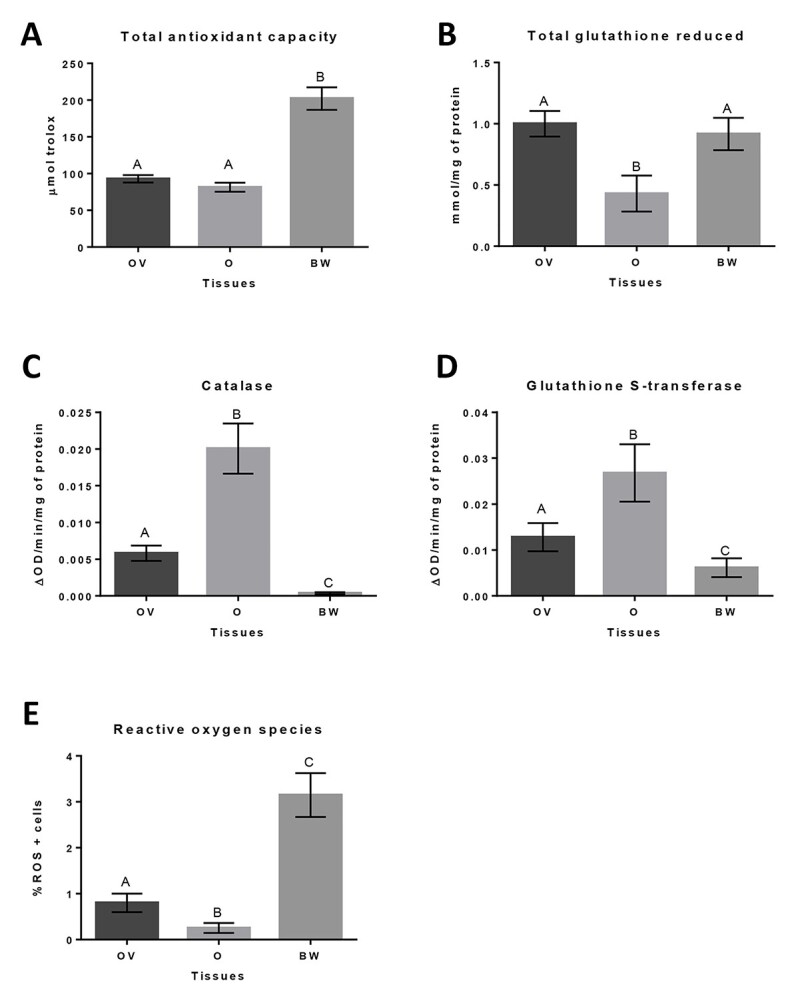
Level of oxidative stress markers (mean ± SD) measured by calorimetric methods: (A) TAC, (B) GSH, (C) CAT, (D) GST, and (E) flow cytometer: (ROS + cells) in different tissues of *E. albidus* (OV, oocytes—O, BW). Different letters indicate significant differences between tissues (Tukey’s multiple comparisons test, *P* ≤ 0.05, *n* = 6, DF = 15).

The subsequent analysis was devoted to determining whether the extensive mitochondrial networks in *E. albidus* germ-line cysts might be impacted increasing levels of cellular stress. Our analysis was based on literature data showing that mitochondria become fragmented and degraded under long-term stress whereas less intense stress or starvation may promote mitochondrial fusion into extensive networks (see Introduction). Extensive mitochondrial networks prompted us to check if, in this case, hyperfusion could also be related to cellular stress. For this purpose, we analyzed the parameters of oxidative stress and the antioxidant cellular defense system in the entire OV (containing germ-line cysts) and the large vitellogenic and individual oocytes (freely floating in the body lumen). To compare the obtained results, we used the BW to represent somatic tissues not connected with oogenesis (Figure 8).

Ovaries and oocytes displayed lower values of TAC measured as TEACaq (μmol Trolox) than the BW. A similar trend was observed for the generation of (ROS + cells). The level of reduced GSH was significantly higher in the OV and BW than in oocytes. Moreover, differences in the levels of oxidative stress markers (CAT and GST) were observed between OV, oocytes, and the BW. The highest activity of both enzymes was detected in oocytes. CAT activity in oocytes was 3.5-fold higher than in the OV and 55-fold higher than in the BW. A similar pattern was observed for GST activity. The level of GST in the oocytes was two times higher than in the OV and 4.5-fold higher than in the BW, in which the GST level was the lowest (Figure 8).

## Discussion

### The stage-specific changes of mitochondria during germ-line cyst oogenesis

Mitochondria are dynamic organelles whose morphology and distribution are changeable and adapt to the cell requirements, changing conditions, and ongoing processes. The dynamism of mitochondria relies on fusion/fission events [58, 74, 75]. The balance between them is reflected in the mitochondrial morphology [38–42, 76–83].

Hoitzing and coauthors [41] proposed a descriptive scale for mitochondrial morphology, based on the degree of the fusion and fission events: (1) static hyperfusion—almost all mitochondria are interconnected into a network, and fission is very rare, (2) dynamic hyperfusion—fusion has the advantage and a mitochondrial network is formed, but fission also occurs, (3) microfused and mesofused mitochondria—fission has the advantage over fusion, and (4) fragmented mitochondria—only individual organelles are visible, and there are no fusion or fission events.

Our observations revealed differences in mitochondrial density and distribution in germ-line cysts at successive stages of their formation and functioning. First of all, as oogenesis progresses, the relative density of mitochondria in the cell cytoplasm slightly increased from 3.0 in the 2-celled cyst to 3.79 in the 16-celled cyst. Additionally, when the cytophore was being formed, mitochondria were being moved to it from cells, and the fully developed cytophore also was equipped with these organelles with the relative density of 2.14.

We observed that in all germ cells and the fully developed cytophore, mitochondria formed extensive aggregates and were connected into large mitochondrial networks. In most cyst cells, individual organelles were also observed, but they represented about 0.5% of the measured total mitochondrial volume only. Based on the classification of Hoitzing [41], this mitochondrial state should be regarded as dynamic hyperfusion. In cysts at advanced oogenesis, the ratio of individual mitochondria increased and reached ~2% in the oocyte and cytophore. Such a small ratio of individual mitochondria may be connected with the ongoing processes of their elimination or selection (discussed next).

However, it was clearly observed that in cysts, extensive mitochondrial networks are formed. Within these networks, hundreds, thousands, or even up to ten thousand individual organelles are fused. Surprisingly, in model organisms as *D. melanogaster*, mouse, and *C. elegans*, although the changes in mitochondrial morphology during oogenesis were described many times, and the mechanisms of fusion and fission events were examined, there is no mention of similar extensive networks [84–88]. In *D. melanogaster*, for example, during germ-line cyst formation and cystocyte divisions, the number of mitochondria increases about eightfold and mitochondrial volume rises from 14- to 20-fold increase [89]. More recent data indicated that a mitochondrial pathway and changes in their morphology is a major mechanism for activation of cell death in *Drosophila* in mid oogenesis. In healthy egg chambers, mitochondrial morphology was defined as extensive tubular networks while degenerating egg chambers (caused by a lack of nutrients) showed clusters of mitochondria. In this case, mitochondrial fission and fusion machinery were responsible for mid-oogenesis cell death [85, 90]. In *C. elegans*, mitochondrial morphology changes in the course of germ cells differentiation. The globular mitochondria predominated in the distal gonad arm, while elongated organelles were more abundant in the proximal arm [88].

According to the literature, there can be a few reasons for such extensive mitochondrial hyperfusion state in *E. albidus* germ-line cysts. Firstly, the mitochondrial networks allow more effective energy production and better distribution of ATP in cell compartments. Indeed, the formation of eggs is a high energy-consuming process; thus, mitochondrial networks in germ cells may intensify ATP production [75, 91]. Moreover, in the studied cysts, only one cell becomes the egg cell; thus, all cells in the cyst work for one oocyte. Therefore, it might be stated that extensive mitochondrial networks found in nurse cells and the cytophore play an essential role in ATP delivery to the growing oocyte.

Secondly, it is believed that mitochondrial networks can keep the mitochondria in good condition and protect them from the effects of mitochondrial damage or mutations [56, 92, 93]. When damage is minor, the fusion of mitochondria may compensate them. When the damage or mutation level is higher, this could lead to the split of such defective mitochondria from the network. In consequence, the fission is a prerequisite for mitochondria elimination by, for example, autophagy (termed in this case mitophagy) [38, 43, 91, 94–97]. In *E. albidus* almost all mitochondria are in the networks, only a small fraction (0.5%) being individual. Moreover, such separated organelles show signs of mitochondrial degeneration (autophagy vesicles). Our interpretation is that severely damaged mitochondria are separated and eventually eliminated. This assumption is in line with literature data showing that mitochondria with mutations or damage are effectively eliminated from cells via autophagy [98–100].

Additionally, as was noted, the highest abundance of individual mitochondria was measured in the cytophore and oocyte from the advanced cysts. It can be explained by the fact that healthy mitochondria are essential for the future egg functioning, and for the proper development of the future generation (as they are inherited almost exclusively from the female line) [101, 102]. Therefore, it seems that mitochondrial selection processes are intensified as oogenesis progresses.

### Mitochondrial activity

As shown in Results section, we observed two kinds of mitochondria in TEM, differing in the mitochondrial matrix density: electron lucent and electron dense. Similarly, electron-dense mitochondria with no obvious cristae were reported in mouse oocytes and zygotes and were regarded as inactive and immature [103]. The two kinds of mitochondria observed in *E. albidus* can be interpreted as a morphological manifestation of the difference in their activity; however, to date, we are not able to determine which of them are active or inactive. Instead, the JC-1 analysis provided a few interesting data. First of all, the activity of mitochondria in germ cells (measured as the ratio of mitochondria with high membrane potential), irrespective of the stage of oogenesis, was always lower than in somatic cells. This agrees with other studies that demonstrated low activity of germ-line mitochondria in the earthworm *Dendrobaena veneta*, *Xenopus*, mouse, and humans [104–106]. This phenomenon can be explained by the need to protect germ cells from damage caused by the side effects associated with high mitochondrial activity, that is, the formation of free radicals and the cellular damage they induce [42, 104–107]. According to the literature, the reduced activity of “germ-line” mitochondria results in a reduced ROS level and is considered by some authors as one way of protecting the next generation against the transmission of deleterious mutations [100, 106]. The second way of protection is the bottleneck phenomenon, which involves segregation and elimination of mitochondria with mtDNA mutations and spreading the undamaged mitochondria in the cytoplasm [100, 108–111]. Analysis in numerous invertebrate and vertebrate species as *Drosophila*, zebrafish, mice, and humans suggest the occurrence of the genetic bottleneck during the maternal transmission of mtDNA (reviewed in [112]). The bottleneck selection can be realized differently; one is on the mitochondrial level. In this case, mitochondria with a high level of deleterious mutations are selected against and removed via mitophagy. Dysfunctional mitochondria can also not replicate their genomes due to the bioenergetic defect. The selection mechanism can also be realized on the cell level. In this case, cells with deleterious mtDNA are also selected against and eliminated, most probably because the mutations lead to cell death, or the bioenergetic defect impairs the capacity for cell division [35, 112–115].

In contrast, despite the lower activity of germ-line mitochondria versus somatic mitochondria, their activity was only moderately lower and peaked at roughly 50%. Looking more precisely, we can discern such a statistically significant difference: within the fully functioning cysts with oocyte and nurse cells, mitochondria in nurse cells were more active, compared to those in the oocytes, in which inactive ones predominated. The general lower mitochondrial activity in the germ-line (vs. somatic line), together with a slight predominance of active mitochondria in nurse cells (vs. oocytes), may reflect the above-discussed mechanism of protecting germ-line mitochondria, especially those in the future egg cell.

Considering the correlation of mitochondrial activity with mitochondrial morphology, several studies indicate that extensive mitochondrial networks can contribute to the higher respiratory activity and increase the bioenergetic capacity in syncytial cysts. Some hypotheses assume that large mitochondrial networks in such cells as striated muscle cells can constitute electrically coupled systems that act as intracellular cables for power transitions [39, 116, 117]. Respiratory chain complexes generate a membrane potential transmitted along mitochondria to oxygen-poor parts of the cell, where the ATP synthase can use it to generate metabolic energy. Mitochondrial fusion and intermixing of mitochondrial content seem crucial for maintaining respiratory capacity in mammalian cells [39, 116, 117]. It is also believed that mitochondrial networks allow organelles to communicate to facilitate access to the products of mtDNA expression or exchange them [38, 81, 116].

In *E. albidus* germ-line cysts, extensive mitochondrial networks, mostly corresponding to up to 1000 fused single mitochondria, seems not to be closely correlated with the acceleration of ATP production. Our results did not strongly support this hypothesis. Instead, it seems more likely that, as in the studies mentioned above [39, 116, 117], mitochondrial networks may improve the energy flow throughout the entire germ-line syncytium, what, in turn, facilitates the oogenesis process.

To sum up, the analysis of the obtained results supports the idea that mitochondrial networks are an efficient way to optimize ATP production and transfer to the places of demand (along with the axis nurse cells–cytophore–oocyte). The formation of extensive mitochondrial networks (dynamic hyperfusion state), significant distribution of the mitochondria in specific regions of the cells, and their abundant accumulation in the cytophore, all act on behalf of one cell—the future egg cell.

### Which one? Selection of the oocyte

Additionally, it is worth paying attention to the observed regularity in the mitochondrial morphology (see below) in the context of determining the developmental fate of germ cells in syncytial cysts. Mechanisms responsible for oocyte determination are well known in some systems, for example, in *D. melanogaster* cysts [10]. Here, the oocyte differentiates from one of the two cells (termed pro-oocytes) containing the highest number (four) of ring canals. Both pro-oocytes enter meiosis but only one of them develops into the oocyte, the second withdraws from meiosis and together with the other 14 cells develops into a nurse cell [118, 119]. It was revealed that the specification of the oocyte takes place much earlier, during cystoblast division, and is maintained until oocyte differentiation. During the first mitotic division, a specific organelle termed the spectrosome segregates asymmetrically and one cell receives the majority of this material [8]. After the final round of mitosis, this “spectrosome-rich” cell will become the oocyte. Moreover, the spectrosome is the precursor of another structure—the fusome, which stretches between ring canals in a cyst. Proteins, mRNA, and organelles move through the ring canals prior to entering the presumptive oocyte. The localization pattern of these factors is critical for the initial specification of the oocyte identity [10, 11, 89, 120].

To date, nothing is known about either factors or mechanisms that determine one cell in *E. albidus* cysts to become an oocyte. In early cysts, all interconnected germ cells are morphologically identical and seem to have the same developmental potential: they and their nuclei are of a similar size, they possess the same kind and arrangement of cell organelles, including structures characteristic for the germ-line such as a nuage material, the density of mitochondria is similar, and as a rule, each cell is connected to the central cytophore by one ring canal. In 16-celled cysts, immediately after the last round of mitotic division, all cells begin meiosis [23]. After that, 15 cells withdraw from meiosis (their nuclei return to interphase) and become nurse cells. Only one cell continues meiosis and develops into an oocyte. The characteristic morphological sign that distinguishes the early oocyte is microvilli formation on its surface [23]. Here, we noted that in early cysts (before the oocyte and nurse cells are morphologically distinguishable) in one cell, the fusion of mitochondria leads to the formation of mitochondrial networks, much larger than the maximal networks formed in their sibling cells. These networks incorporated up to 10 000 single mitochondria (see cell no. 3 in 4-celled and 16-celled cysts in Table 2), while in the other 15 cells, the maximal number of connected mitochondria was lower than 1000. This observation allows us to hypothesize that this particular cell may develop into an oocyte in the future. This extensive network itself does not determine the oocyte and is probably only a very early expression of its determination. It should also be strongly emphasized that these are preliminary observations and assumptions, and there is a need for further research to elucidate the mechanism of oocyte specification in an annelid cyst equipped with a cytophore.

### Antioxidant capacity in syncytial oogenesis

As mentioned above, the process of egg formation is energy-consuming, and mitochondria have to deliver the necessary amount of energy. However, energy production is directly linked with the formation of ROS—in the sense that the more ATP there is, the more ROS are produced. ROS are generated during mitochondrial oxidative metabolism, or they may arise from interactions with exogenous sources, such as xenobiotics, ionizing radiation, cytokines, or bacteria [121, 122]. Briefly, ROS are necessary for the proper functioning of cell metabolism, cell signaling, cellular responses, as well as apoptosis [94, 111]. When ROS occur in excess, whether due to an increase in their production or a decrease in the cellular antioxidant capacity, oxidative stress occurs. Oxidative stress, in turn, results in direct or indirect ROS-mediated damage of cellular lipids, proteins, or DNA, thus inhibiting signal transduction pathways and normal cellular functions [121, 123].

In oogenesis, there is competition between the production and supply of sufficient energy for egg production and protection of that egg cell (and thus a future embryo) against damage of the cellular components. Oxidative stress during oogenesis can influence future generations’ quality and viability, as was shown, inter alia, in mouse oocytes and zygotes [42, 103]. Additionally, in the vast majority of metazoans, mitochondria are inherited by the maternal route only (mitochondria accumulated in the oocyte during oogenesis constitute a pool of embryonic mitochondria after fertilization) [101, 102]. The good quality of mitochondria is thus crucial for the health and proper development of the embryo [35, 36]. The oxidative metabolism of mitochondria on the one hand, and their good quality on the other, seem to be opposed to each other [37, 58–60].

In connection with the potential danger of ROS formation, cells and tissues have developed a variety of defense mechanisms to neutralize the harmful effects of free radicals [123]. Here, we have measured the oxidative stress and antioxidant capacity in syncytial oogenesis. However, it should be clarified that, due to the requirements in the protocols of the available test methods, it was not possible to analyze particular germ-line cysts at the successive stages of oogenesis (as was performed for mitochondrial morphology, distribution, and activity). However, we focused on testing the antioxidant mechanisms in the entire OV, consisting of several syncytial cysts, and in individual oocytes, freely floating in the body lumen. For comparison, we selected a somatic structure—the BW—that represented a sample not related to oogenesis and sexual reproduction.

Surprisingly, our analysis revealed that germ-line cysts and oocytes have a lower level of ROS and a less effective oxidative defense system (TAC) than the BW cells. However, the level of reduced GSH, which is involved in the defensive role against ROS acting in distinct pathways [124], was at the same level in OV and the BW, and only in oocytes was significantly lower. On the other hand, the activity of the oxidative stress markers CAT and GST was higher in oocytes than in germ-line cysts, whereas the lowest level was noted in the BW. GST plays a role in the metabolism and intracellular transport of metabolites [125, 126]. GST catalyzes the conjugation of reactive electrophiles with GSH, with the potential of forming reactive intermediates, in particular when GSH levels in the cells are attenuated [125, 126]. GST represents an intracellular protective mechanism against ROS [124]. On the other hand, CAT is a crucial antioxidant enzyme and acts by destroying cellular hydrogen peroxide to produce water and oxygen [127]. These together indicate a mechanism of the induction of antioxidant enzymes and the powerful antioxidant potential within germ-line cysts, especially in large vitellogenic oocytes of *E. albidus*. Thus, it is an essential line of defense against oxidative stress, which seems legitimate and may protect future gametes against the harmful effects of free radicals.

Suppose we try to combine these results with the large mitochondrial networks in *E. albidus* syncytial cysts. In that case, it emerges as an efficient, at least two-way defense mechanism that protects oogenesis and future egg cells. Although the studies devoted to changes in mitochondrial morphology under the various conditions are complicated and challenging to interpret due to many variable factors and stress conditions, and the results may depend on the type of cells and species, some similarities can arise. Generally speaking, mitochondria fuse and form an extended network to maintain homeostasis, when cells are subjected to mild stress (including UV irradiation, inhibition of RNA transcription, some drugs that inhibit cytosolic protein synthesis, and moderate nutrient starvation). They resist mitophagy and increase ATP production to adjust to cellular stresses [82, 92, 98]. However, in the case of severe stress, mitochondria become fragmented. It leads to the elimination of the mitochondria via mitophagy or, if stress is prolonged, via apoptosis ([39, 82, 92, 98] and references therein). The cellular response to the mild stresses occurs in a process termed stress-induced mitochondrial hyperfusion, in which the mitochondria are highly interconnected. The hyperfusion response requires the fusion machinery proteins, as Mitofusin (MFN) 1 and OPtic Atrophy (OPA) 1, and other important factors [92].

Experimental data also suggest that when the mechanism responsible for mitochondrial fusion is deficient, it contributes to the mitochondrial fragmentation observed in different forms of mitochondrial dysfunction, as was shown in cardiac myocytes, rat kidney proximal tubular cells, and neurons [92]. Mutations in genes encoding proteins responsible for fusion and fission processes cause severe pathologies, significantly correlated with muscles, brain, and nervous system symptoms. One of the intensively studied processes linked with aberration in mitochondrial fusion is neurodegeneration.

Many other experimental studies have reported the dependence between changes in ROS levels and mitochondrial morphology. Although the genetic mechanisms were different, as a rule, high ROS levels induced mitochondrial fragmentation, whereas low ROS levels promoted mitochondrial fusion and thus formation of networks through acting on mitochondrial fusion and fission proteins [94, 128]. In *D. melanogaster*, mutations in OPA1 (an essential protein involved in mitochondrial fusion) induced aberrations in mitochondrial morphology and increased the production of ROS and susceptibility to oxidative stressors [129]. In *E. albidus*, ROS level was low in OV and oocytes, in which the hyperfused mitochondria were also described. These results agree with the above data and suggest that mitochondrial fusion protects against oxidative stress [78, 128, 129]. Altogether these data indicate that the modulation of mitochondrial fusion/fission machinery maintains metabolic homeostasis during stress conditions. Contrary to the abovementioned examples, mitochondrial fusion is reduced in cancer cells, and fission and fragmented mitochondria are documented. Researchers believe it is related to several phenotypes of cancer cells, such as cell cycle progression and resistance to apoptosis [92]. It is worth noting that some anticancer drugs such as mitogen-activated protein kinase (MAPK)- or PI3K-inhibitors cause mitochondrial fusion and form an extensive network [92].

Moreover, it should be noted that many aspects of the function of mitochondrial fission and fusion remain unclear. One of a mitochondrial network’s proposed and well-proven functions is to provide antioxidative protection. It is believed that mitochondrial networks prevent cell death and mitophagy, while severely damaged mitochondria are eventually eliminated from the network [38, 43, 58, 94, 95, 128, 130, 131]. Antioxidative defense systems in germ-line cysts and oocytes of *E. albidus*, together with extensive mitochondrial networks, seem to constitute an effective mechanism protecting mitochondria from damage. Consequently, eliminating the significantly damaged organelles via autophagy may play a supporting role, as observed in *E. albidus*, in which only rare autophagosomes with remnants of cell organelles (including mitochondria) were found during syncytial oogenesis.

## Conclusions

(1) In germ-line cysts at all analyzed stages of their formation and functioning, 98–99.5% of mitochondria were fused into mitochondrial networks.
(2) The most extensive mitochondrial networks consisted of 10 000 connected mitochondria.
(3) Individual mitochondria are scattered among fused ones. Some of them undergo degradation, suggesting that damaged mitochondria are removed from networks.
(4) One cell in forming cysts has a more extensive mitochondrial network than the maximal networks formed in its sibling cells. It may be the early morphological expression of oocyte determination.
(5) Mitochondria in germ-line cysts, irrespective of the stage of cyst functioning, were always less active than in somatic cells. Moreover, germ-line mitochondria in nurse cells and the cytophore were more active than those in the growing oocytes.
(6) Analysis of TAC, level of GSH, and the oxidative stress markers CAT and GST revealed differences between germ-line cysts and oocytes and the somatic structures (the BW).
(7) Antioxidative defense systems in germ-line cysts and oocytes of *E. albidus*, together with extensive mitochondrial networks, can effectively protect mitochondria from damage.

## Supplementary Material

Suppl_1_ioac035

Suppl_2_ioac035

Suppl_3_ioac035

Suppl_4_ioac035

Suppl_5_ioac035

Suppl_6_ioac035

## Data Availability

The data underlying this article will be shared on reasonable request to the corresponding author.
