# Supplementary figures and images for: All for one: changes in mitochondrial morphology and activity during syncytial oogenesis
Source: Biol Reprod. 2022 Feb 14;106(6):1232–53. doi: 10.1093/biolre/ioac035 (PMC9720109; doi:10.1093/biolre/ioac035)

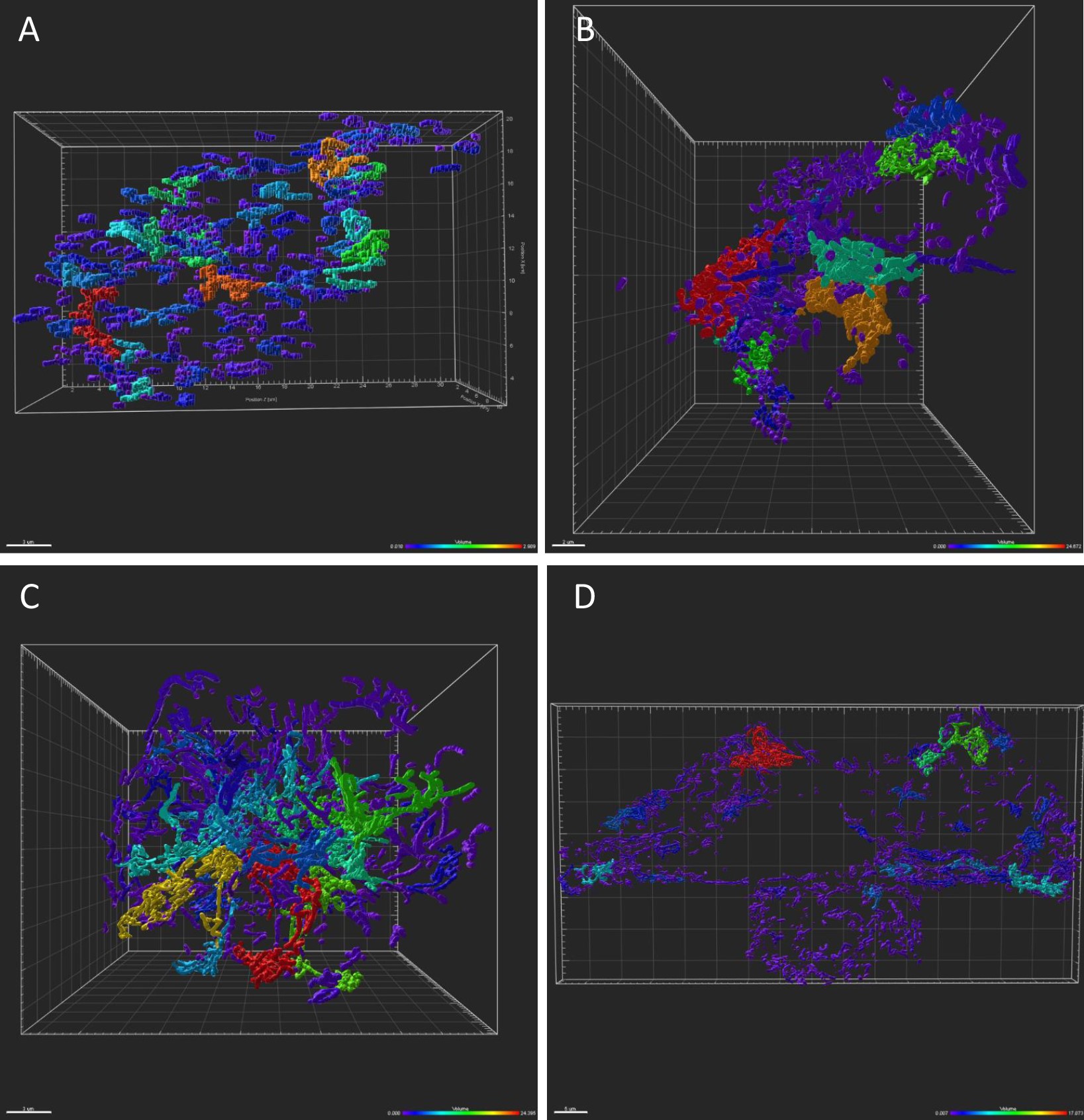

Supplement: Suppl_6_ioac035 [file suppl_6_ioac035.jpeg]
